# Rpl5-Inducible Mouse Model for Studying Diamond-Blackfan Anemia

**DOI:** 10.15190/d.2019.9

**Published:** 2019-09-30

**Authors:** Shideh Kazerounian, Daniel Yuan, Matthew S. Alexander, Alan H. Beggs, Hanna T. Gazda

**Affiliations:** Boston Children's Hospital, Division of Genetics and Genomics, The Manton Center for Orphan Disease Research, Boston, MA, USA; Harvard Medical School, Boston, MA, USA; University of Alabama at Birmingham and Children’s of Alabama, Departments of Pediatrics and Genetics, Division of Neurology, Birmingham, AL, USA

**Keywords:** Diamond-Blackfan anemia, Ribosomal Protein L5, Rpl5-Inducible Mouse Model.

## Abstract

Diamond-Blackfan anemia (DBA) is a rare congenital bone marrow disorder with mutations in ribosomal protein genes. Several animal models have been developed to study the pathological mechanism of DBA. Previously, we reported that the complete knock-out of both Rpl5 and Rps24 alleles were lethal, while heterozygous Rpl5+/- and Rps24+/- mice showed normal phenotype.  To establish a more efficient mouse model for mimicking DBA symptoms, we have taken advantage of RNAi technology to generate an inducible mouse model utilizing tetracycline-induced down-regulation of Rpl5.    After two weeks of treatment with doxycycline in drinking water, a subset of treated shRNA Rpl5+/- adult mice developed mild anemia while control mice had normal complete blood counts. Similarly, treated shRNA Rpl5+/- mice developed reticulocytopenia and bone marrow erythroblastopenia. Detection of DBA symptoms in these mice make them a valuable DBA model for studying the pathological mechanism underlying DBA and for further assessment of the disease and drug testing for novel therapies.

Diamond-Blackfan anemia (DBA) is a rare congenital bone marrow disorder inherited in an autosomal dominant pattern and resulting from haploinsufficiency of ribosomal proteins. It is characterized by macrocytic anemia, reticulocytopenia, and bone marrow erythroblastopenia as well as congenital malformations in about 50% of patients^1^.

The first major breakthrough in the molecular pathogenesis of DBA came from the discovery of the ribosomal protein S19 gene^2^, followed by identification of mutations in 24 additional ribosomal protein (RP) genes including RPL5^3^. Additionally, mutations in two non-ribosomal protein genes, GATA1, encoding a critical transcription factor for red blood cell maturation, and TSR2, encoding a pre-RNA processing protein, have been reported in a subset of patients^4-6^. Furthermore, we reported a significant decrease in the expression level of GATA1 protein but not mRNA in primary hematopoietic cells from patients with mutations in RP genes^6^.

To address pathological mechanisms underlying DBA, several animal models have been generated. Homozygous Rps19-/- mice are embryonic lethal while heterozygotes had similar levels of RPS19 protein and mRNA as wild-type litermates^7^. We have reported that knocking-out Rpl5 and Rps24 alleles also leads to embryonic lethality, while heterozygous Rpl5+/- and Rps24+/- mice showed normal phenotypes at birth and throughout their development with no detectable differences between the expression levels of RPL5 and RPS24 mRNA and protein compared to those of wild-type mice^8^. Interestingly, a small number of these mice developed soft tissue sarcomas, also seen in some of patients with DBA^8^. Due to the severity of symptoms associated with RPL5 mutations in patients, we decided to focus our studies on the molecular mechanism of RPL5 deficiency induced postnataly^3^. Here, we report the generation of a conditional Rpl5 mouse line using an RNA interference (RNAi) approach. All animal studies were approved by Boston Children's Hospital's Institutional Animal Care and Use Committee.

A validated short-hairpin RNA (shRNA) construct targeting the mouse Rpl5 mRNA transcript was cloned into a TRE3G-based *Col1A1*-targeting vector (pColA1-TRE3G-GFP-miR30) and co-electroporated into pre-engineered KH2 embryonic stem cells, which contain a reverse tet-transactivator (rtTA) cassette integrated into the *Rosa26* locus^9^. In parallel, we obtained an shRNA-luciferase (shRNA *Renilla*,**shRNA* Ren*) mouse line to use as a non-specific shRNA control line. Both mouse lines were generated by Mirimus Laboratories (Mirimus Inc., Brooklyn, NY) on the C57BL/6 background.

To assess the effects of *Rpl5 *down-regulation, mice either heterozygous or homozygous for the shRNA at the ColA1 and Rosa26 loci were generated. To increase the tetracycline effect for a stronger mRNA knockdown, we used mice that were heterozygous for *ColA1* and homozygous for* Rosa26, *which would increase the production of reverse tetracycline transactivator and therefore, the effect of tetracycline treatment (as described above). Henceforth, we will refer to shRNA *Rpl5*^*+/-*^mice as shRNA *Rpl5* and shRNA* Ren*^*+/- *^as control mice. Five to eight-week old female and male shRNA *Rpl5*mice were treated with 2mg/mL of doxycycline in drinking water^10^. After 2 weeks of treatment, a mild anemia was detected in about 20% of the treated shRNA *Rpl5 *mice while control**mice were normal (**Table 1**). Further hematological studies revealed marked reticulocytopenia in all treated shRNA *Rpl5* mice (n=3) (reticulocytes 1.77%, 0.1x10^6^/ul) versus control**mice (n=3) (reticulocytes 3.8%, 0.24x10^6^/ul) and bone marrow erythroblastopenia (myeloid to erythroid linage 4.9 in all shRNA *Rpl5 *mice and 3.4 in control mice). To further investigate erythropoiesis in shRNA *Rpl5 *and control mice, methylcellulose colony assays^11^ were performed on bone marrow cells isolated from these mice to quantify the number of burst-forming unit- erythroid BFU-E and colony forming unit-erythroid CFU-E colonies as well as colony forming-unit granulocyte macrophage (CFU-GM). Our results showed a decrease in the number of BFU-E and CFU-E colonies in shRNA *Rpl5 *mice compared to control, which correlates to a decrease in the proliferation level of erythroid progenitor cells and a slight decrease of CFU-GM colonies (**Figure 1A**). We next performed flow cytometry on freshly isolated bone marrow cells from shRNA *Rpl5**and* control**mice to compare the percentage of differentiated erythroid cells in each cell population. In our experiment, we examined two erythroid populations: less mature CD71^high^Ter119^med^ and more mature CD71^high^Ter119^high^^12^. shRNA *Rpl5 *mice had lower numbers of CD71^high^Ter119^med^ (0.6% vs. 0.9%) and CD71^high^Ter119^high^ (3.2% vs. 5.3%) cells as compared to control mice (**Figure 1B**). GATA-1 is a necessary factor for the survival and terminal differentiation of erythroid progenitors^13^.

**Figure 1:**
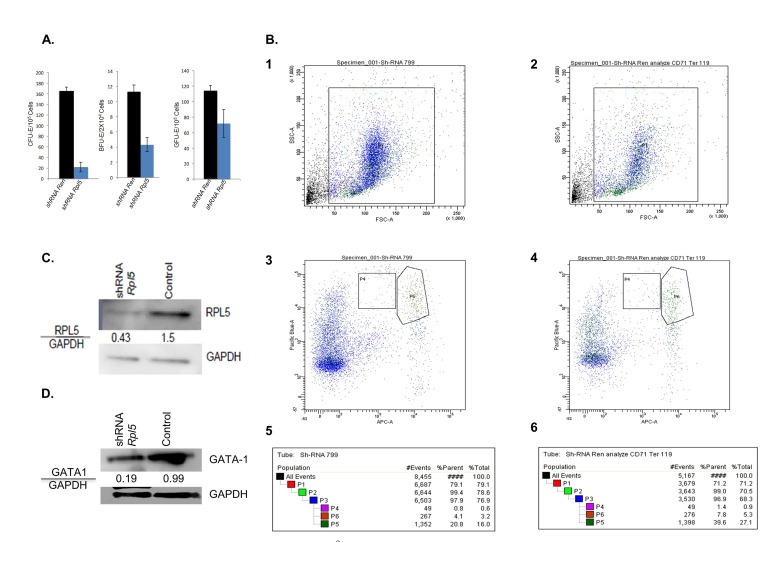
**A. **Methylcellulose colony assay on bone marrow cells from doxycycline treated shRNA Rpl5 and control mice (n=3), (CFU-E, P=0.0003; BFU-E, P=1; GFU-GM, P=0.3); **B. **FACS analysis of bone marrow cells from shRNA Rpl5 and control mice. Total bone marrow cells from shRNA Rpl5 (B.1) and control (B.2) mice; viable cells were gated for analysis and sorting. We used the cell-surface markers CD71 (Y-axis) and Ter119 (X-axis) to separate mature erythroids and erythroid precursors. The most mature erythroblasts are CD71lowTer119high, while the erythroid precursors are CD71highTer119intermediate cells. In our FACS analysis the gated population comprises CD71highTer119med cells (P4) and CD71highTer119high cells (P5) from shRNA Rpl5 (B.3 and B.5) and control (B4 and B6) mice; the results here are collected from one mouse per group and are representative of 6 independent experiments; in each experiment, cells were collected from 1-3 mice for each group and sorted independently; **C. **Western Blot analysis of RPL5 protein expressions in CD71highTer119high cells. There was a significant decrease in the expression level of RPL5 in the treated shRNA Rpl5 compared to the control mice; **D. **Western Blot analysis of GATA1 protein expressions in CD71highTer119high cells. There was a significant decrease in the expression of GATA1 in the shRNA Rpl5 group compared to the control mice. In these experiments, GAPDH was used as a loading control, and they are representative of 3 independent experiments.

**Table 1:** Complete blood count in shRNA Rpl5 and shRNA Ren mice treated with doxycycline RBC: red blood cells (P=0.13); Hb: hemoglobin (P=0.0008); HTC: Hematocrit (P=0.117); MCV: corpuscular volume (P=0.3); WBC: white blood cell (P=0.46); NE: neutrophils (P=0.34); LY: lymphocytes (P=0.27); MO: monocytes (P=0.15); EO: eosinophils (P=0.45); BA: basophils (P=0.15); PLT: platelet count (P=0.92). Graphpad T test calculator website has been used to determine the P values based on standard division.

Parameter (Units)	shRNA Rpl5 (Mean Result, n=3)	shRNA Ren (Mean Results, n=3)	Normal Range
RBC (M/µL)	5.7 ­±0.4	8.3­±1.3	6.36-9.42
Hb (g/dL)	5.9±0.3	11.3±0.5	11.0-15.1
HTC (%)	26.8±1.9	41.8±7.3	35.1-45.4
MCV (fl)	47.3±0.9	50.3±2.3	45.4-60.3
WBC (K/µL)	6.0±2.7	12.6±7.6	1.8-10.7
NE (K/µL)	1.0±0.2	4.2±3.0	0.1-2.4
LY (K/µL)	2.9±0.8	7.4±3.4	0.9-9.3
MO (K/µL)	0.07±0.005	0.54±0.66	0.0-0.4
EO (K/µL)	0.02±0.01	0.38±0.43	0.0-0.2
BA (K/µL)	0.01±0.0	0.17±0.09	0.0-0.2
PLT (K/µL)	674.3±292.3	626.3±349.7	592-2972

To assess expression levels of RPL5 and GATA1 proteins in shRNA *Rpl5*mice, western blots were performed on CD71^high^Ter119^high^ cell population total cell lysates using anti-RPL5 (Novus Biologic, NBP1-31413) or anti-GATA-1 antibodies (Sc-1234)^8^. These results demonstrated a significant decrease in expression of both RPL5 (**Figure 1C**) and GATA1 (**Figure 1D**) in cells from the treated shRNA *Rpl5*mice compared to control mice. On the other hand, quantitative PCR on two sorted erythroid populations showed similar levels of *Gata1 *mRNA expression in shRNA *Rpl5*and control mice (data not shown), as was shown in human cells^6^. The reduction of GATA1 protein is likely due to the reduction of RPL5, impaired ribosomal erythropoiesis, lower numbers of ribosomes, and altered GATA1 translation, as reported in human cells^6,14^.

In summary, we have generated and characterized a novel DBA mouse model, which allows an inducible and graded down-regulation of *RpL5* gene expression. These mice recapitulate the major features of DBA including anemia, reticulocytopenia, and bone marrow erythroblastopenia^1^. Therefore, these shRNA *Rpl5^+/-^*mice may provide an effective approach for studying DBA and testing novel therapies.

## References

[1] Li Hojun, Lodish Harvey F., Sieff Colin A. (2018). Critical Issues in Diamond-Blackfan Anemia and Prospects for Novel Treatment. Hematology/Oncology Clinics of North America.

[2] Draptchinskaia Natalia, Gustavsson Peter, Andersson Björn, Pettersson Monica, Willig Thiébaut-Noël, Dianzani Irma, Ball Sarah, Tchernia Gil, Klar Joakim, Matsson Hans, Tentler Dimitri, Mohandas Narla, Carlsson Birgit, Dahl Niklas (1999). The gene encoding ribosomal protein S19 is mutated in Diamond-Blackfan anaemia. Nature Genetics.

[3] Ulirsch JC, Verboon JM, Kazerounian S, Guo MH, Yuan D, Ludwig LS (2018). The Genetic Landscape of Diamond-Blackfan Anemia. Am J Hum Genet..

[4] Sankaran Vijay G., Ghazvinian Roxanne, Do Ron, Thiru Prathapan, Vergilio Jo-Anne, Beggs Alan H., Sieff Colin A., Orkin Stuart H., Nathan David G., Lander Eric S., Gazda Hanna T. (2012). Exome sequencing identifies GATA1 mutations resulting in Diamond-Blackfan anemia. Journal of Clinical Investigation.

[5] Gripp Karen W., Curry Cynthia, Olney Ann Haskins, Sandoval Claudio, Fisher Jamie, Chong Jessica Xiao-Ling, Pilchman Lisa, Sahraoui Rebecca, Stabley Deborah L., Sol-Church Katia (2014). Diamond-Blackfan anemia with mandibulofacial dystostosis is heterogeneous, including the novel DBA genesTSR2andRPS28. American Journal of Medical Genetics Part A.

[6] Ludwig Leif S, Gazda Hanna T, Eng Jennifer C, Eichhorn Stephen W, Thiru Prathapan, Ghazvinian Roxanne, George Tracy I, Gotlib Jason R, Beggs Alan H, Sieff Colin A, Lodish Harvey F, Lander Eric S, Sankaran Vijay G (2014). Altered translation of GATA1 in Diamond-Blackfan anemia. Nature Medicine.

[7] Matsson H., Davey E. J., Draptchinskaia N., Hamaguchi I., Ooka A., Leveen P., Forsberg E., Karlsson S., Dahl N. (2004). Targeted Disruption of the Ribosomal Protein S19 Gene Is Lethal Prior to Implantation. Molecular and Cellular Biology.

[8] Kazerounian Shideh, Ciarlini Pedro D.S.C., Yuan Daniel, Ghazvinian Roxanne, Alberich-Jorda Meritxell, Joshi Mugdha, Zhang Hong, Beggs Alan H., Gazda Hanna T. (2016). Development of Soft Tissue Sarcomas in Ribosomal Proteins L5 and S24 Heterozygous Mice. Journal of Cancer.

[9] Premsrirut Prem K., Dow Lukas E., Kim Sang Yong, Camiolo Matthew, Malone Colin D., Miething Cornelius, Scuoppo Claudio, Zuber Johannes, Dickins Ross A., Kogan Scott C., Shroyer Kenneth R., Sordella Raffaella, Hannon Gregory J., Lowe Scott W. (2011). A Rapid and Scalable System for Studying Gene Function in Mice Using Conditional RNA Interference. Cell.

[10] Jaako Pekka, Flygare Johan, Olsson Karin, Quere Ronan, Ehinger Mats, Henson Adrianna, Ellis Steven, Schambach Axel, Baum Christopher, Richter Johan, Larsson Jonas, Bryder David, Karlsson Stefan (2011). Mice with ribosomal protein S19 deficiency develop bone marrow failure and symptoms like patients with Diamond-Blackfan anemia. Blood.

[11] Gazda Hanna T., Kho Alvin T., Sanoudou Despina, Zaucha Jan M., Kohane Isaac S., Sieff Colin A., Beggs Alan H. (2006). Defective Ribosomal Protein Gene Expression Alters Transcription, Translation, Apoptosis, and Oncogenic Pathways in Diamond-Blackfan Anemia. Stem Cells.

[12] Koulnis Miroslav, Pop Ramona, Porpiglia Ermelinda, Shearstone Jeffrey R., Hidalgo Daniel, Socolovsky Merav (2011). Identification and Analysis of Mouse Erythroid Progenitors using the CD71/TER119 Flow-cytometric Assay. Journal of Visualized Experiments.

[13] Pevny Larysa, Simon M. Celeste, Robertson Elizabeth, Klein William H., Tsai Shih-Feng, D'Agati Vivette, Orkin Stuart H., Costantini Frank (1991). Erythroid differentiation in chimaeric mice blocked by a targeted mutation in the gene for transcription factor GATA-1. Nature.

[14] Khajuria Rajiv K., Munschauer Mathias, Ulirsch Jacob C., Fiorini Claudia, Ludwig Leif S., McFarland Sean K., Abdulhay Nour J., Specht Harrison, Keshishian Hasmik, Mani D.R., Jovanovic Marko, Ellis Steven R., Fulco Charles P., Engreitz Jesse M., Schütz Sabina, Lian John, Gripp Karen W., Weinberg Olga K., Pinkus Geraldine S., Gehrke Lee, Regev Aviv, Lander Eric S., Gazda Hanna T., Lee Winston Y., Panse Vikram G., Carr Steven A., Sankaran Vijay G. (2018). Ribosome Levels Selectively Regulate Translation and Lineage Commitment in Human Hematopoiesis. Cell.

